# The Association Between Anemia and Depressive Symptoms in Non-White Male Adults: National Health and Nutrition Examination Survey (2005–2018)

**DOI:** 10.34172/jrhs.2023.133

**Published:** 2023-12-29

**Authors:** Jinsong Mou, Haishan Zhou, Zhangui Feng

**Affiliations:** ^1^Pingshan District Maternal and Child Healthcare Hospital of Shenzhen, Shenzhen, China

**Keywords:** Anemia, Depression, Hemoglobin, Nonlinearity, American, PHQ-9

## Abstract

**Background:** The relationship between anemia and depression remains controversial. This study aimed to investigate the association between hemoglobin (Hb) levels and depressive symptoms.

**Study Design:** A cross-sectional study.

**Methods:** This study was conducted using National Health and Nutrition Examination Survey data from 2005–2018. Hb levels were obtained from laboratory files, and depressive symptoms were assessed using the Patient Health Questionnaire (PHQ-9). Multivariable logistic regression analysis and smoothing plots were performed to examine the relationship between anemia and depression, including potential nonlinear associations.

**Results:** The study included 6008 male adults. Multivariable analysis revealed that anemia was associated with an increased odds ratio for mild (OR=1.49, 95% CI: 1.06, 2.10) and moderate (OR=2.05, 95% CI: 1.14-3.70) anemia. Additionally, each additional g/dL of Hb was significantly inversely associated with developing depression (OR=0.91, 95% CI: 0.85, 0.96). A nonlinear relationship was detected between Hb and depression, with an inflection point at 15 g/dL. Below this threshold, there was a significantly negative association between Hb and depression (OR=0.88, 95% CI: 0.79, 0.98); no significant relationship was observed above it (OR=1.05, 95% CI: 0.84, 1.31).

**Conclusion:** Anemia was positively associated with depression in non-White American men. A nonlinear relationship between Hb and depression was detected, and it had a saturation effect. A significant negative correlation with depression was observed when the Hb level was below 15 g/dL.

## Background

 Depression is a prevalent mental disorder affecting approximately 300 million people globally.^1^ In 2019, around 18.5% of American adults had symptoms of depression, with 15.0% being men.^2^ The exact cause of depression is not fully understood but involves factors such as epigenetic modifications,^3^ neurotransmitter imbalances,^4^ inflammation,^5^ and abnormal neuroplasticity.^6^

 Anemia is characterized by insufficient red blood cells to meet the body’s needs. The prevalence of anemia in the United States increased from 4.03% to 6.49% between 1999 and 2020.^7^ Anemia affects both physical health and mental health, resulting in decreased physical functioning and neuropsychological development,^8,9^ as well as fatigue and emotional/psychological problems.^10^ Anemia might also induce changes in the hippocampus, corpus striatum, and monoamine levels that lead to mental disorders,^11^ increasing the risk of mental health. Similarly, depression may play a reciprocal role in anemia.^12^

 The relationship between these two disorders is still a topic of controversy. Some studies^13,14^ have reported that anemia was frequently accompanied by depressive symptoms in older adults, while a meta-analysis has shown a positive association between anemia and depression in adults and maternal populations.^15^ In a case-control study, individuals with depression were found to have lower hemoglobin (Hb) levels.^16^ However, other studies have not reported conclusive evidence supporting the link between anemia and depression.^17,18^ Therefore, whether anemia is a risk factor for depression needs further elucidation.

 Several studies have investigated the association between anemia and depression among specific populations, such as women,^19,20^ elderly individuals,^21^ cancer patients,^22^ children and adolescents,^23^ and healthy adults.^24^ However, research on the association between anemia and depression among men is scarce, and some studies have reported controversial outcomes in this regard. Yi et al^25^ discovered a link between lower serum ferritin levels and depressive symptoms in middle-aged Japanese men, while other studies conducted on Korean populations demonstrated no association in this respect.^26,27^ Due to the presence of nonspecific symptoms, men with depression often face challenges in obtaining an accurate diagnosis^28,29^ and are frequently overlooked.^30^ Furthermore, men with depression are more likely to commit suicide.^31^ A meta-analysis has also concluded that depression in men should not be disregarded.^32^ Similarly, men with anemia may also be at risk of being overlooked, as their symptoms may be less apparent. Consequently, the potential association between anemia and depression in men may be easily missed. Therefore, it is crucial to explore the link between anemia and depression in men. González et al^33^ found that non-white individuals experience significantly higher depression chronicity and lower depression care use and guidelines. In addition, research has shown that non-white individuals are often underdiagnosed or undertreated for depression due to factors, including socioeconomic disparities, access to appropriate care, and patient-physician relationships.^34^ Consequently, non-white males have been selected as the primary population of interest for this study.

 However, research investigating the association between Hb levels and depression among non-white male adults in the United States is currently limited. Therefore, this study has attempted to examine the relationship between Hb levels and depression, specifically within this population.

## Methods

###  Study Population

 The National Health and Nutrition Examination Survey (NHANES) is a cross-sectional study of the Centers for Disease Control and Prevention conducted by the National Center for Health Statistics (NCHS) that provides nationally representative estimates of the US population. An analysis was performed based on NHANES data from 2005 to 2018. All NHANES data and information are publicly available at https://www.cdc.gov/nchs/nhanes/index.htm.

 In accordance with the research objective and due to a limited number of participants with severe anemia and from other races, NHANES participants were excluded from our study based on the exclusion criteria; they included (1) PHQ-9, anemia, and other covariates with missing data, (2) non-Hispanic White and other races, (3) females, and (4) participants diagnosed with severe anemia. Finally, 6008 participants were included in the study.

###  Ascertainment of Depression and Anemia

 The Patient Health Questionnaire-9 (PHQ-9) depression scale was used for the diagnosis of depression and was administered during a face-to-face mobile examination center (MEC) interview to assess depression symptoms over the last two weeks. The scale has nine items, and each item is scored from 0 (not at all) to 3 (nearly every day). Total scores range from 0 to 27, and adults with scores of 0–4 are considered to have no symptoms of depression, while those with scores of ≥ 5 are considered to have depression symptoms.^35^

 The amount of Hb (g/dL) was obtained from the laboratory file. Anemia was defined based on the World Health Organization’s (WHO’s) cutoff points for Hb. The Hb in male participants (15 years of age and older) was defined as no anemia, mild, and moderate anemia according to Hb levels ≥ 13 g/dL, 11–12.9 g/dL, and 8–10.9 g/dL, respectively.^36^

###  Covariates 

 The covariates considered known or potential influences on the association between anemia and depression were chosen through a review of the literature and input from healthcare experts. Sociodemographic variables, lifestyle habits, socioeconomic status indicators, medical history, and dietary patterns were among the covariates chosen for inclusion in the study. Specifically, these variables comprised age, ethnicity, education, citizenship, smoking status, marital status, family size, family income to poverty ratio, alcohol consumption, body mass index (BMI, kg/m^2^), hypertension (HTN), hyperlipidemia, and asthma, as well as intake of fruits, vegetables, grains, dairy products, meat, and eggs.

 The study utilized various criteria to categorize participants. The family income to poverty ratio (PIR) was calculated by dividing the family income by the survey year-specific poverty guidelines, accounting for variations in family size and geographic location.^37^ PIR was employed to establish low (PIR < 1.3) and mild-high (PIR ≥ 1.3) categories. The WHO classification was used to divide participants into underweight ( < 18.5 kg/m^2^), normal weight (18.5–24.9 kg/m^2^), overweight (25.0–29.9 kg/m^2^), and obese ( ≥ 30 kg/m^2^) groups based on their BMI^38^. HTN was defined as having a systolic blood pressure ≥ 140 mm Hg and/or diastolic blood pressure ≥ 90 mm Hg^39^. Hyperlipidemia was characterized as an elevated cholesterol level (total cholesterol ≥ 200 mg/dL [5.18 mmol/L], triglyceride level ( ≥ 150 mg/dL), low-density lipoprotein ≥ 130 mg/dL [3.37 mmol/L], high-density lipoprotein < 40 mg/dL [1.04 mmol/L], or the use of cholesterol-lowering agents.^40^ Asthma was ascertained through the question, “Ever been told you have asthma or use anti-asthmatic drugs”.

###  Statistical Analyses

 The information was recorded in a database using Excel and analyzed by a statistical package of the survey in R software. Continuous variables were expressed as means with their standard errors. Categorical variables were presented as proportions and frequencies. Weighted univariable and multivariable logistic regressions were performed to examine the association between anemia and depression. We built three sets of models, adjusting for different covariates, to verify the stability of the results. The crude model controlled for no variables. Model one controlled for age, ethnicity, education, citizenship, total number of people in the family, marital status, and family income-to-poverty ratio. Model II controlled for all variables in Model I plus smoking, drinking, BMI, HTN, hyperlipidemia, and asthma. Confounding factors were selected based on prior literature and a significance level of *P* < 0.05. If nonlinearity was found between anemia and depression, two-piecewise logistic regression models were constructed on both sides of the inflection point. Finally, a subgroup analysis of the association between anemia and depression was performed in terms of age, ethnicity, education, and PIR. The *P* values were two-sided, and a *P* value ≤ 0.05 was considered statistically significant.

## Results

###  Baseline Characteristics of Participants

 A total of 6008 male individuals from NHANES (2005–2018) were included in this study, with 34.92% Mexican Americans, 46.01% non-Hispanic Blacks, and 19.07% other Hispanics. There were 1152 (19.17%) individuals with depression. The baseline characteristics of the study participants based on anemia are presented in Table 1. Among all participants, the distribution of education, family income to poverty ratio, hyperlipidemia, and egg intake showed no significant differences by anemia level (*P*> 0.05). Comparing different groups (Table 1), the distribution of anemia was significantly different by age, ethnicity, citizenship, smoking, marital status, total number of people in the family, drinking, BMI, HTN, asthma, depression, fruit, vegetables, grains, dairy, and meat intake (all *P* ≤ 0.05).

**Table 1:** Baseline characteristics of participants, NHANES 2005-2018, weighted

**Categorical Variables**	**Without anemia**		**Mild anemia**		**Moderate anemia**		***P***** value**
	**Number**	**Percent**	**Number**	**Percent**	**Number**	**Percent**	
Age (y)							0.001
20-39	2099	51.38	34	13.72	2	5.84	
40-59	1886	36.01	109	39.40	17	41.36	
≥ 60	1540	12.61	273	46.88	48	52.81	
Ethnicity							0.001
Mexican American	2021	38.85	65	15.08	12	13.70	
Non-Hispanic Black	2407	38.83	308	74.95	49	76.52	
Other Hispanic	1097	22.32	43	9.96	6	9.79	
Educational level							0.815
< High school	1951	31.70	139	30.41	29	38.78	
High school	1369	26.64	113	27.71	13	21.09	
> High school	2205	41.67	164	41.88	25	40.13	
Citizenship							0.001
Citizen by birth or naturalization	4176	73.07	392	93.18	59	88.82	
Not a citizen of the US	1349	26.93	24	6.82	8	11.18	
Smoking							0.001
Never	2674	53.27	169	44.31	28	42.76	
Former	1450	21.73	148	31.03	23	33.61	
Current	1401	25.01	99	24.66	16	23.63	
Marital status							0.001
Married	2888	49.25	219	51.16	39	55.71	
Widowed	154	1.56	45	8.96	6	6.59	
Divorced	467	6.88	53	11.65	8	11.95	
Separated	229	3.89	22	4.43	5	8.11	
Never married	1144	24.78	54	16.97	5	11.77	
Unmarried cohabitation	643	13.64	23	6.83	4	5.88	
Total number of people in the family							0.001
1	1142	19.81	131	30.96	18	25.49	
2	1120	16.70	111	24.30	18	28.99	
3	922	17.67	70	16.34	12	21.11	
4	924	18.71	48	13.14	7	11.81	
5	687	13.67	34	10.16	6	6.16	
6	384	7.48	12	2.89	2	2.47	
≥ 7	346	5.96	10	2.21	4	3.98	
Family income to poverty ratio							0.793
Low	1917	34.60	161	36.32	23	32.32	
Mid-high	3608	65.40	255	63.68	44	67.68	
Drinking							0.001
Never	403	7.36	40	9.96	13	20.50	
Former	895	12.51	146	28.63	20	28.14	
Mild	1806	31.51	132	32.77	26	40.86	
Moderate	748	14.29	36	9.83	4	4.65	
Heavy	1673	34.33	62	18.80	4	5.85	
Body mass index (kg/m^2^)							0.001
Normal weight	1191	21.61	133	31.62	26	38.49	
Underweight	59	1.02	7	1.76	1	3.41	
Overweight	2134	38.05	127	30.96	17	27.71	
Obese	2141	39.33	149	35.67	23	30.39	
Hypertension							0.001
No	3267	67.08	128	37.11	16	28.95	
Yes	2258	32.92	288	62.89	51	71.05	
Hyperlipidemia							0.958
No	1824	36.32	138	36.15	22	34.28	
Yes	3701	63.68	278	63.85	45	65.72	
Asthma							0.050
No	4904	88.47	356	84.29	55	84.89	
Yes	621	11.53	60	15.71	12	15.11	
Depression							0.001
No	4500	81.35	311	73.69	45	69.10	
Yes	1025	18.65	105	26.31	22	30.90	
Continuous variables	**Mean**	**SE**	**Mean**	**SE**	**Mean**	**SE**	***P*** **-value**
Fruit intake [ce/day, mean (SE)]	1.04	0.03	0.96	0.08	0.63	0.11	0.002
Vegetables intake [ce/day, mean (SE)]	1.53	0.02	1.36	0.06	1.21	0.16	0.006
Grains intake [oe/day, mean (SE)]	8.15	0.11	6.74	0.22	6.30	0.53	0.001
Dairy intake [ce/day, mean (SE)]	1.51	0.03	1.13	0.06	1.46	0.43	0.001
Meat intake [oe/day, mean (SE)]	6.57	0.07	5.88	0.24	4.65	0.44	0.001
Eggs intake [oe/day, mean (SE)]	0.74	0.02	0.62	0.05	0.65	0.15	0.066

*Note*. Ce: Cup equivalents; oe: Ounce equivalents; NHANES: National Health and Nutrition Examination Survey, SE: standard deviation

###  Factors Associated with Depression

 Univariate analysis was applied to the available data, demonstrating that the factors in terms of age, other Hispanics, high school education, a non-citizen of the United States, marital separation, family with two or more than seven number of people, former, mild and moderate drinking, overweight, obesity, hyperlipidemia, fruit, grain, dairy, and meat intake were not significantly related to depression (all* P* > 0.05). However, mild (OR = 1.56, 95% CI: 1.16, 2.09) or moderate anemia (OR = 1.95, 95% CI: 1.14, 3.32), non-Hispanic Black (OR = 1.18, 95% CI: 1.00, 1.39), and former (OR = 1.35, 95% CI: 1.11, 1.64) or current (OR = 2.01, 95% CI: 1.67, 2.41) smoking represented a positive association with depression. Moreover, widowed (OR = 2.18, 95% CI: 1.46, 3.26), divorced (OR = 1.98, 95% CI: 1.55, 2.52), never married (OR = 1.75, 95% CI: 1.46, 2.08), unmarried cohabitation (OR = 1.52, 95% CI: 1.20, 1.92), heavy drinking (OR = 1.52, 95% CI: 1.08, 2.12), underweight (OR = 1.84, 95% CI: 1.04, 3.26), HTN (OR = 1.33, 95% CI: 1.18, 1.51), and asthma (OR = 1.80, 95% CI: 1.48, 2.19) were positively associated with depression. On the other hand, lower Hb (OR = 0.92, 95% CI: 0.86, 0.97), education more than high school (OR = 0.73, 95% CI: 0.63, 0.84), mild-high family income (OR = 0.63, 95% CI: 0.54, 0.73), and vegetable intake (OR = 0.92, 95% CI: 0.87, 0.97) were negatively associated with depression. In addition, being in families with three (OR = 0.75, 95% CI: 0.59, 0.95), four (OR = 0.75, 95% CI: 0.58, 0.96), five (OR = 0.70, 95% CI: 0.55, 0.89), and six (OR = 0.70, 95% CI: 0.53, 0.94) children had a negative relationship with depression (Table 2).

**Table 2:** The results of the univariate analysis of depression

**Variables**	**Number**	**Percentage**	**OR (95% CI)**	***P***** value**
Anemia				
Non-Anemia	5525	91.96	1.00	
Mild	416	6.92	1.56 (1.16, 2.09)	0.004
Moderate	67	1.12	1.95 (1.14, 3.32)	0.015
Hemoglobin (g/dL)	-		0.92 (0.86, 0.97)	0.004
Age (y)				
20-39	2135	35.54	1.00	
40-59	2012	33.49	1.16 (0.98, 1.37)	0.094
≥ 60	1861	30.98	1.02 (0.87, 1.20)	0.802
Ethnicity				
Mexican American	2098	34.92	1.00	
Non-Hispanic Black	2764	46.01	1.18 (1.00, 1.39)	0.049
Other Hispanic	1146	19.07	1.24 (0.96, 1.60)	0.095
Educational level				
< High school	2119	35.27	1.00	
High school	1495	24.88	0.93 (0.77, 1.11)	0.406
> High school	2394	39.85	0.73 (0.63, 0.84)	0.001
Citizenship				
Citizen by birth or naturalization	4627	77.01	1.00	
Not a citizen of the US	1381	22.99	0.90 (0.74, 1.09)	0.270
Smoking				
Never	2871	47.79	1.00	
Former	1621	26.98	1.35 (1.11, 1.64)	0.003
Current	1516	25.23	2.01 (1.67, 2.41)	0.001
Marital status				
Married	3146	52.36	1.00	
Widowed	205	3.41	2.18 (1.46, 3.26)	0.001
Divorced	528	8.79	1.98 (1.55, 2.52)	0.001
Separated	256	4.26	1.43 (0.93, 2.19)	0.100
Never married	1203	20.02	1.75 (1.46, 2.08)	0.001
Unmarried cohabitation	670	11.15	1.52 (1.20, 1.92)	0.001
Total number of people in the family				
1	1291	21.49	1.00	
2	1249	20.79	0.92 (0.72, 1.16)	0.463
3	1004	16.71	0.75 (0.59, 0.95)	0.020
4	979	16.29	0.75 (0.58, 0.96)	0.025
5	727	12.1	0.70 (0.55, 0.89)	0.005
6	398	6.62	0.70 (0.53, 0.94)	0.017
≥ 7	360	5.99	0.81 (0.59, 1.10)	0.174
Family income to poverty ratio				
Low	2101	34.97	1.00	
Mid-high	3907	65.03	0.63 (0.54, 0.73)	0.001
Drinking				
Never	456	7.59	1.00	
Former	1061	17.66	1.30 (0.91, 1.86)	0.154
Mild	1964	32.69	1.03 (0.75, 1.42)	0.850
Moderate	788	13.12	1.35 (0.94, 1.94)	0.107
Heavy	1739	28.94	1.52 (1.08, 2.12)	0.016
Body mass index				
Normal weight	1350	22.47	1.00	
Underweight	67	1.12	1.84 (1.04, 3.26)	0.036
Overweight	2278	37.92	0.89 (0.73, 1.09)	0.273
Obese	2313	38.5	1.01 (0.85, 1.21)	0.886
Hypertension				
No	3411	56.77	1.00	
Yes	2597	43.23	1.33 (1.18, 1.51)	0.001
Hyperlipidemia				
No	1984	33.02	1.00	
Yes	4024	66.98	0.99 (0.87, 1.14)	0.921
Asthma				
No	5315	88.47	1.00	
Yes	693	11.53	1.80 (1.48, 2.19)	0.001
Fruit intake (ce/day)	-	-	0.99 (0.94, 1.04)	0.675
Vegetables intake (ce/day)	-	-	0.92 (0.87, 0.97)	0.005
Grains intake (oe/day)	-	-	0.99 (0.98, 1.01)	0.272
Dairy intake (ce/day)	-	-	0.99 (0.95, 1.04)	0.780
Meat intake (oe/day)	-	-	0.99 (0.98, 1.01)	0.251
Eggs intake (oe/day)	-	-	0.93 (0.87, 1.00)	0.067

*Note*. ce: Cup equivalents; oe: Ounce equivalents; CI: Confidence interval; OR: Odds ratio.

###  Association Between Anemia and Depression

 Three models were constructed using the binary logistic regression model to investigate the association between anemia and depression (Table 3). In the unadjusted model and Model I, which minimally adjusted for several variables, each 1 g/L increment in Hb was significantly inversely associated with developing depression (non-adjusted: OR = 0.92, 95% CI: 0.87, 0.97; model I: OR = 0.92, 95% CI: 0.86, 0.98). Furthermore, Model II, which fully adjusted for all variables, revealed the inverse relationship between Hb and depression (OR = 0.91, 95% CI: 0.85, 0.96). The results were statistically significant.

**Table 3:** Multivariable analysis of the association between anemia and depression

**Variables**	**Non-adjusted**		**Model Ⅰ**		**Model Ⅱ**	
	**OR (95% CI)**	***P *****value**	**OR (95% CI)**	***P *****value**	**OR (95% CI)**	***P *****value**
Hemoglobin (g/dL)	0.92 (0.86, 0.97)	0.004	0.92 (0.86, 0.98)	0.01	0.91 (0.85, 0.96)	0.002
Anemia						
Non-anemia	1.00		1.00		1.00	
Mild anemia	1.56 (1.16, 2.09)	0.004	1.51 (1.09, 2.10)	0.014	1.49 (1.06, 2.10)	0.022
Moderate anemia	1.95 (1.14, 3.32)	0.015	1.98 (1.09, 3.62)	0.026	2.05 (1.14, 3.70)	0.017
*P* for trend	0.001		0.003		0.003	

*Note*. CI: Confidence interval; OR: Odds ratio; BMI: Body mass index.Model I was adjusted for age, ethnicity, education, citizenship, total number of people in the family, marital status, and family income to poverty ratio. Model II was adjusted for age, ethnicity, education, citizenship, total number of people in the family, marital status, family income to poverty ratio, smoking, drinking, BMI, hypertension, hyperlipidemia, asthma, fruit, vegetables, grains, dairy, meat, and eggs intake.

 Consistent results were also found when Hb was converted from a continuous variable to a categorical variable, including normal, mild, and moderate anemia (Table 3). The results of the crude model without adjusting for any factors showed that mild and moderate anemia were positively associated with depression (mild: OR = 1.56, 95% CI: 1.16, 2.09; moderate: OR = 1.95, 95% CI: 1.14, 3.32). In the minimally adjusted model I, there was a positive relationship between anemia and depression (mild: OR = 1.51, 95% CI: 1.09, 2.10; moderate: OR = 1.98, 95% CI: 1.09, 3.62). Model II, adjusted for all variables, still demonstrated a positive connection between anemia and depression (mild: OR = 1.49, 95% CI: 1.06, 2.10; moderate: OR = 2.05, 95% CI: 1.14, 3.70). The association between anemia and depression in the three models and the increasing trend of the effect sizes in different groups were statistically significant.

###  Nonlinear Link Between Anemia and Depression

**Figure 1:**
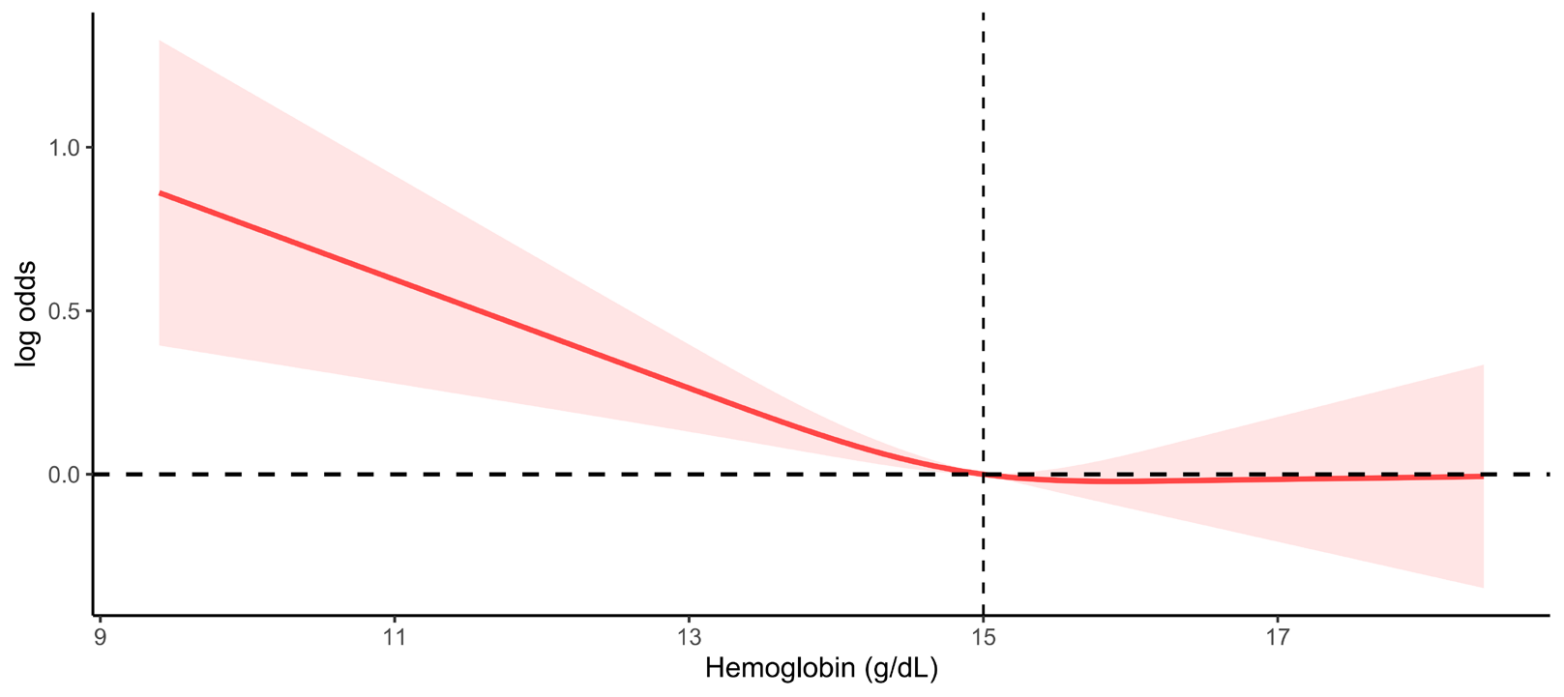
The nonlinear relationship between anemia and depression

 Based on the results (Figure 1), the association between Hb and the risk of depression was nonlinear. The inflection point for Hb was 15 g/dL. When Hb was on the left side of this inflection point ( ≤ 15 g/dL), a significantly negative association was found between Hb and depression (OR = 0.88, 95% CI: 0.79, 0.98, *P* = 0.018). However, when Hb was on the right side of this inflection point ( > 15 g/dL), no relationship was observed between anemia and depression (OR = 1.05, 95% CI: 0.84, 1.31, *P* = 0.687).

###  Subgroup Analysis Results

 The subgroup analysis revealed a significant association between mild or moderate anemia and depression in certain groups. Specifically, young adults aged 20–29 years old with mild anemia had a higher odds ratio for depression (OR = 2.46, 95% CI: 1.17, 5.16), while individuals aged 40–59 years showed a slightly lower OR (OR = 1.64, 95% CI: 1.01, 2.66). Individuals over 60 years with moderate anemia were more strongly associated with depression (OR = 2.59, 95% CI: 1.47, 4.57). Mexican-Americans demonstrated a significant association between mild (OR = 2.28, 95% CI: 1.22, 4.25) and moderate (OR = 4.12, 95% CI: 1.26, 13.46) anemia and depression. Non-Hispanic Blacks were also found to have a significant association between moderate anemia and depression (OR = 1.91, 95% CI: 0.99, 3.67).

 The analysis represented a significant association between mild anemia and depression among individuals with less than high school education (OR = 1.73, 95% CI: 1.10, 2.74), more than high school education (OR = 1.53, 95% CI: 1.02, 2.30), and mild-high family income (OR = 1.65, 95% CI: 1.16, 2.34). In addition, the results showed that none of the interactions were observed based on the analyzed variables (*P* for interaction > 0.05 for all covariates, Table 4).

**Table 4:** Subgroup analyses of the association between anemia and depression

**Variables**	**Mild anemia**		**Moderate anemia**		***P***** interaction**
	**OR (95%CI)**	***P *****value**	**OR (95%CI)**	***P *****value**	
Age (y)					0.271
20-29	2.46 (1.17, 5.16)	0.018	6.43 (0.39, 106.72)	0.192	
40-59	1.64 (1.01, 2.66)	0.045	1.08 (0.37, 3.16)	0.888	
≥ 60	1.29 (0.89, 1.86)	0.175	2.59 (1.47, 4.57)	0.001	
Ethnicity					0.183
Mexican American	2.28 (1.22, 4.25)	0.010	4.12 (1.26, 13.46)	0.020	
Non-Hispanic Black	1.36 (0.96, 1.94)	0.081	1.91 (0.99, 3.67)	0.053	
Other Hispanic	1.77 (0.78, 4.04)	0.173	0.31 (0.03, 2.76)	0.288	
Education					0.878
< High school	1.73 (1.10, 2.74)	0.019	1.47 (0.65, 3.29)	0.350	
High school	1.42(0.84, 2.41)	0.187	2.79 (0.83, 9.39)	0.097	
> High school	1.53 (1.02, 2.30)	0.041	2.07 (0.76, 5.62)	0.152	
Family income to poverty ratio					0.822
Low	1.42 (0.93, 2.17)	0.102	2.10 (0.88, 5.00)	0.091	
Mid-high	1.65 (1.16, 2.34)	0.006	1.92 (0.90, 4.12)	0.093	

*Note*. CI: Confidence interval; OR: Odds ratio.

## Discussion

 Our study findings confirmed that anemia was significantly associated with vulnerability to depression, and a significant relationship was identified between Hb level and depression. Additionally, a nonlinear relationship was observed between Hb and depression. This study provides valuable insights, as this specific population’s health concerns may often be overlooked. These findings indicate that prioritizing and addressing the health problems of these specific subgroups are critical for reducing barriers to mental health care and depression burden. Further, our research can provide reference data that can inform government policies aimed at preventing and controlling anemia and depression in this population.

 Anemia is probably associated with greater fatigue, leading to a worse quality of life and decreased emotional wellbeing^41^. To assess the potential relationship between anemia and depression, a multivariable analysis was conducted to adjust for covariates. The results revealed that each additional g/dL of Hb was inversely associated with developing depression (OR = 0.91). Categorizing Hb levels, a positive relationship was found between anemia and depression. The OR for mild and moderate anemia was 1.49 and 2.05, respectively. This positive effect was also evident in subgroup analyses based on various factors. Several potential reasons may explain the observed relationship between anemia and depression. People with anemia may experience reduced physical function or oxygen delivery to the brain,^8^ as well as alterations in neurotransmitter systems,^9,10^ all of which potentially contribute to an increased risk of depression. Furthermore, anemia can cause changes in the hippocampus, corpus striatum, and monoamine levels that may lead to depression.^11^ Several meta-analyses have indicated a significant association between anemia and depression. One meta-analysis of older adults represented that anemia was associated with worse cognitive function and depression symptoms,^42^ while another meta-analysis in adults reported a significant correlation between low Hb levels and depression (OR/RR = 1.43).^15^ Hence, anemia might be related to vulnerability to depression. However, a Korean study demonstrated no significant association between anemia and depression in men.^27^ The reasons for these discrepant findings may reflect differences in the study design (e.g., cross-sectional vs. cohort), variations in depression screening criteria, or the unique sociodemographic characteristics of the Korean population (e.g., distinct economic levels, living environments, cultural backgrounds, and lifestyles).

 In the present study, a significantly increasing trend effect size was observed between mild-to-moderate anemia and depression among subjects in the fully adjusted model (*P* for trend = 0.003). Meanwhile, when Hb was ≤ 15 g/dL, a significantly negative association was identified between Hb level and depression (OR = 0.88). This result is consistent with the findings of a cross-sectional analysis in which depression was independently associated with lower Hb levels (*β* = -0.074; *P* = 0.05).^43^ To some extent, anemia might affect the brain and mood; thus, correcting anemia may be beneficial in improving the oxygen supply to brain tissue and reducing the risk of cognitive dysfunction and depression symptoms.^8,9,44^

 Our findings revealed no significant association between anemia and depression when Hb was > 15 g/dL (OR = 1.05, 95% CI: 0.84, 1.31). Vulser et al^24^ also reported no association between depression status and high Hb levels ( > 16 g/dL in men). Similarly, Lever-van Milligen et al^18^ noted that the association between higher Hb ( > 16.1 g/dL in men) and increased depression severity did not reach a significance level after adjusting for socio-demographics, disease indicators, and lifestyle. However, further academic research is needed to understand the lack of association when Hb is at a high level.

 Our study has some limitations. Firstly, the cross-sectional nature of the study precluded us from definitively establishing a causal relationship between anemia and depression. Secondly, the extent of generalization is limited as the majority of participants were Mexican, Black, and Hispanic-American men without severe anemia. Thirdly, reporting bias may have been reported by some participants who had hidden sensitive information during the survey. Lastly, some uncontrolled variables affecting the results may not have been considered confounders due to missing data or sample size. Future research could improve upon the limitations of our study using longitudinal designs to establish causal links, including broader ranges of populations and disease severity, reducing reporting bias through a variety of methods, and considering more potential confounders. These improvements could strengthen the validity of future findings and provide more reliable insights into the relationship between anemia and depression.

**Highlights**

- The present study is the first to report a significant association between anemia and depression in non-White American men.
- The relationship between mild or moderate anemia and depression was statistically significant, with a trend of increasing effect sizes.
- A nonlinear association was observed between hemoglobin (Hb) and depression, with an inflection point of 15 g/dL.
- A significant negative association was found between Hb and depression for levels of 15 g/dL or below, while no relationship was observed for levels above 15 g/dL.

## Conclusion

 Overall, the present study, to the best of our knowledge, is the first to report a significant association between anemia and depression in non-White American men. The results showed that the relationship between mild or moderate anemia and depression was statistically significant, with a trend of increasing effect sizes. Additionally, a nonlinear association was observed between Hb and depression. Our findings highlight the importance of continuous screening for these diseases and the need for a comprehensive approach to healthcare that addresses both physical and mental health concerns. Such a conclusion would suggest that further research is necessary to explore intervention strategies, confirm their causal relationship, and evaluate the possible mechanisms through large-scale, multicenter longitudinal follow-up studies.

## Acknowledgements

 We would like to thank the participants for their cooperation in the NHANES project and the staff members for contributing to data collection and making the data publicly available.

## Authors’ Contribution

**Conceptualization:** Jinsong Mou.

**Data curation:** Haishan Zhou.

**Formal analysis:** Haishan Zhou, Zhangui Feng.

**Funding acquisition:** Jinsong Mou.

**Investigation:** Jinsong Mou, Haishan Zhou.

**Methodology:** Jinsong Mou.

**Project administration:** Jinsong Mou.

**Resources:** Jinsong Mou.

**Software:** Jinsong Mou.

**Supervision:** Jinsong Mou.

**Validation:** Haishan Zhou, Jinsong Mou, Zhangui Feng.

**Visualization:** Jinsong Mou and Haishan Zhou.

**Writing–original draft:** Jinsong Mou, Haishan Zhou, Zhangui Feng.

**Writing–review & editing:** Jinsong Mou.

## Competing Interests

 The authors declare that they have no competing interests.

## Ethical Approval

 The NHANES program was approved by the NCHS Ethics Review Board, and all participants provided written informed consent.

## Funding

 The Shenzhen Pingshan Anemia Prevention and Control Programme and Shenzhen Pingshan Healthcare Research Project [202183] supported the study.
